# Design and preliminary evaluation of a VR-based optokinetic stimuli system for vestibular rehabilitation: insights from clinical end users

**DOI:** 10.3389/fmed.2026.1766706

**Published:** 2026-03-04

**Authors:** Korak Sarkar, Kathleen Delpy, Summer Skelton, Alec Slayden, Nicole R. Villemarette-Pittman

**Affiliations:** 1 Ochsner Clinic Foundation, New Orleans, LA, United States; 2 Ochsner Neuroscience Institute, New Orleans, LA, United States; 3 Ochsner Academics & Research, New Orleans, LA, United States; 4 Ochsner Biodesign Lab, New Orleans, LA, United States; 5 Physical Medicine and Rehabilitation Service Southeast Louisiana Veterans Health Care System, New Orleans, LA, United States; 6 University of Queensland School of Medicine, Brisbane, QLD, Australia; 7 Xavier Ochsner College of Medicine, New Orleans, LA, United States

**Keywords:** medical extended reality, neurology, physical therapy, vestibular, virtual reality

## Abstract

**Background:**

Vestibular therapy is essential for treating dizziness and balance disorders. There is growing evidence supporting the benefits of incorporating Virtual Reality (VR) into vestibular therapies.

**Objective:**

To gather feedback from neuro-rehabilitation specialists on an optokinetic virtual environment (OVE).

**Methods:**

An OVE modeled after the traditional optokinetic drum was developed for commercially available VR headsets and tested by practicing vestibular therapists. Participants completed a Likert-based survey and semi-structured interviews to assess their perceptions of the OVE. Qualitative data were analyzed thematically.

**Results:**

Ten therapists with an average of 9 years of clinical experience (range: 3–15 years) participated from October 2024 to March 2025. The likelihood of using the OVE in clinical practice was rated highly, with Likert scores ranging from 6 to10 (median = 9), where 10 indicated a high likelihood. Five key themes emerged for potential improvements: (1) Clinical Usability and Setup, (2) Control and Customization, (3) Immersive Design and Realism, (4) Output and Measurement Preferences, and (5) Implementation Barriers.

**Conclusion:**

Vestibular rehabilitation clinicians expressed strong interest in utilizing VR-based optokinetic stimuli. Feedback from this study will inform iterative, user-focused application improvements. Future studies will test the improved OVE with patients to evaluate tolerance, efficacy, and usability.

## 1 Introduction

Neurological diseases impact 1 in 3 humans and are currently the leading cause of global disability (1, 2). Conditions impacting the nervous system encompass a diverse set of pathologies like epilepsy, traumatic brain injury, cerebrovascular disease, neuro-infectious, neuro-degenerative and autoimmune processes, as well as balance disorders. The global burden of neurological disease is large and is expected to significantly increase by the year 2050 (3). There exists robust evidence validating the integral role of neurorehabilitation to augment emerging and targeted treatments for specific diseases (4–6). Specialized physical, occupation, and speech therapy facilitate recovery and functional optimization for many neurological conditions, like stroke (7, 8), Parkinson’s (9), dementia (10), multiple sclerosis (11), balance disorders, and related falls (12).

Despite evidence supporting the efficacy of relatively low-cost neuro-rehabilitation interventions, access to neurorehabilitation worldwide remains limited and is amplified by barriers to accessing an already scarce resource (13, 14). This issue is particularly stark in under-resourced rural and urban areas in the United States as well as low- and middle-income countries (15, 16). Public health and humanitarian crises like the COVID-19 pandemic and climate-related disasters have and will continue to increase the burden of neurological disease and aggravate the lack of access to neurorehabilitation worldwide (17, 18). These current and future realities of neurological disease burden highlight the need for accessible, ruggedized, and equitable neurorehabilitation. There is growing evidence that technologies like extended reality (XR) can help address the large and growing need for rehabilitation by augmenting existing clinical infrastructure (19).

Disorders of the vestibular system lead to functional impairments in balance and are often associated with the symptom of dizziness (20). Approximately 1 in 10 American adults will experience dizziness each year, and adults who report dizziness have a higher mortality rate, independent of age, race, gender, and cardiovascular risk factors (21).

Specialized physical and occupational therapies facilitate recovery and optimize function for many neurological conditions, including balance disorders and related falls (12). Vestibular physical therapy and exercise have been proven to be integral to the treatment of vestibular pathology (22, 23). Vestibular rehabilitation incorporates individualized visual, balance, and mobility exercises based on the type of impairment (23). Inclusion of optokinetic stimuli, like moving patterns that engage the visual and vestibular systems, into vestibular rehabilitation is utilized to gradually desensitize patients to movements and contextual factors which provoke dizziness (24–26). Integrating optokinetic stimuli can help patients tolerate complex and busy environments necessary for community reintegration, such as driving and shopping (27).

**FIGURE 1:**
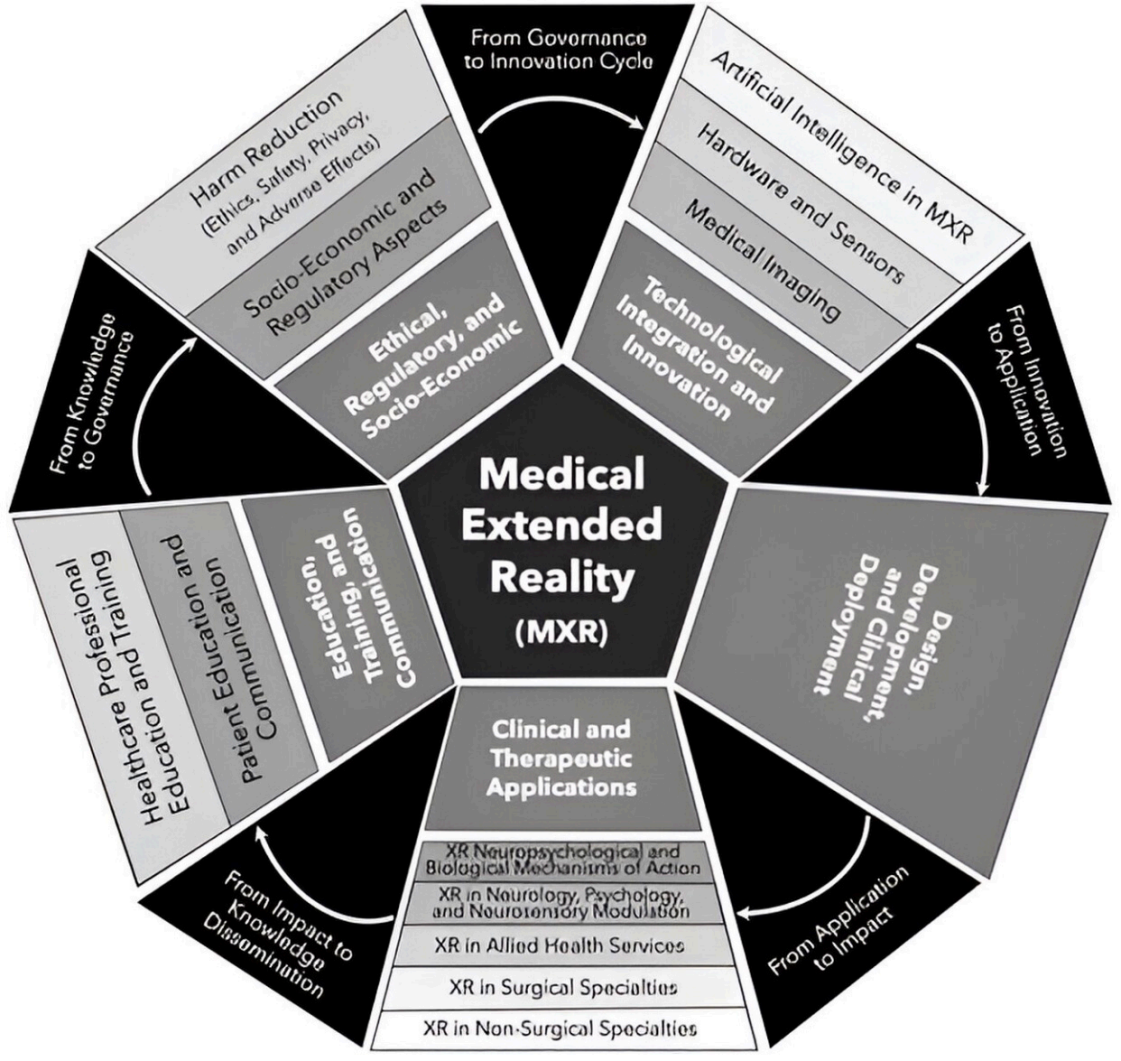
MXR Taxonomy encompassing 5 broad domains that encompass 13 primary topics.

Virtual and Augmented Reality (VR and AR, respectively) are technologies that are increasingly being used in healthcare (28). VR completely immerses users in a digital environment while (AR juxtaposes a virtual image, i.e., a hologram, in a real-life setting. Mixed reality blends AR and VR to create real-time, hybrid physical and digital environments (29). The recent release of consumer-grade VR computing head-mounted displays (HMDs), i.e., headsets, has expedited the adoption of VR in a diverse array of fields including healthcare. This rapidly evolving area has been recognized by the United States Food and Drug Administration (FDA) and is referred to as medical extended reality (MXR) (28, 30) (Figure 1).

The VR Clinical Outcomes Research Experts (VR-CORE) model recommends a phased development and validation approach for MXR interventions analogous to the FDA’s Phase I-III pharmacotherapy development paradigm (31). In this approach, the creation of MXR interventions progresses through 3 stages named VR1, VR2, and VR3. VR1 focuses on gathering end-user feedback and applying human-centered design principles to create an MXR intervention. This intervention is then assessed for usability, acceptance, and potential adverse consequences in VR2. Finally, VR3 assesses the efficacy of the intervention through randomized controlled trials analogous to a pivotal trial in drug and medical device development.

There is growing but limited evidence supporting the general use of MXR interventions in neurorehabilitation, and specifically for balance disorders (32–35). The current body of evidence, however, remains modest. Moreover, the evidence is limited by its heterogenous inclusion criteria, MXR intervention approach, comparator design, and clinical outcomes assessment. There remains a gap in knowledge about the usability and efficacy of MXR neurorehabilitation interventions. There is a need to better characterize the optimal design of an MXR intervention for neurorehabilitation that will be accepted by patients, operationalized by clinicians, and scalable for modern healthcare delivery.

The purpose of this project is to gather feedback from neurorehabilitation specialists on an optokinetic virtual environment (OVE) prototype developed for commercially available VR HMDs. The primary objective of this VR1-phase study is to examine clinician willingness to adopt an VR–based OVE for vestibular rehabilitation and to identify perceived barriers to its routine clinical use. Specifically, this study aims to characterize how vestibular rehabilitation clinicians perceive the usability, perceived clinical value, and workflow compatibility of an optokinetic virtual environment (OVE). Additionally, this study intends to understand logistical constraints that may limit implementation of VR solutions as well as functionality that would promote use in neurorehabilitation.

## 2 Materials and methods

### 2.1 Ethical consideration

Ethics approval for this study was provided by the Ochsner Health Institutional Review Board (2024.054). Informed consent was waived for clinician participants. All interview data collected for this study were fully de-identified and anonymized prior to analysis to ensure participant privacy and confidentiality. Secure storage protocols were implemented in compliance with institutional and federal data protection standards. Access to the data was restricted to authorized study personnel directly involved in data collection and analysis. No compensation was provided to study participants. All procedures were conducted in accordance with appropriate ethical guidelines and applicable regulations. All data and materials generated in this study were managed in accordance with the FAIR (Findable, Accessible, Interoperable, and Reusable) data principles, ensuring that de-identified qualitative transcripts, analysis, and related materials can be made available upon reasonable request to promote transparency and reproducibility.

### 2.2 Development

An HMD-based VR optokinetic drum was developed using a human-centered design approach as described in the VR-CORE Model (31). This paper reports results from the VR1 stage, the first of the three phases of clinical design, focusing on eliciting feedback from end-user neurorehabilitation clinicians. The OVE was developed by a multidisciplinary team including a neurorehabilitation physician (KS), neuro-physical therapist (KD), an MXR developer (AS), and a biopsychologist (NVP).

**FIGURE 2:**
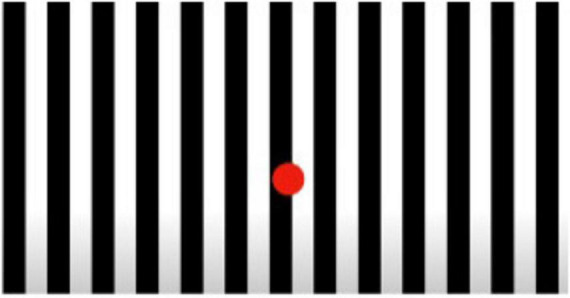
Optokinetic stimuli.

The Academy of Neurologic Physical Therapy of the American Physical Therapy Association’s recent update to clinical practice guidelines for vestibular rehabilitation recommends habitation exercises whereby individuals are systematically exposed to provocative stimuli that, with repeated exposures, reduces symptomology (23). Optokinetic stimuli are specifically recommended as a tool for habituation and visual motion desensitization. Additionally, optokinetic stimuli challenges the visual system therefore improving integration with vestibular and somatosensory inputs. Finally, optokinetic stimuli can enable contextual adaptation to visually complex and realistic environment. This approach supports improved tolerance of motion-rich setting encountered in daily life and informed the development of the OVE prototype (Figure 2).

Unity, a real-time 3D engine, served as the platform for development, facilitating the creation of an immersive virtual environment. GitHub, a code-hosting platform, aided in development and version control. The MXR developer designed and created dynamic VR optokinetic stimulus tailored for neurorehabilitation. A Meta Quest 3 HMD was used to display the optokinetic environment.

### 2.3 Recruitment

The study recruited 10 neurorehabilitation clinicians from 5 different clinics with experience and interest in vestibular rehabilitation. The sample size is consistent with the design of similar studies and the general recommendations for qualitative analysis (36–38). To be included in the study, clinicians were required to be physical or occupational therapists with > 2 years of experience treating patients with vestibular disease. Individuals who were unable to tolerate the HMD or OVE exposure were excluded. Ten clinicians met criteria and were offered a chance to participate. One clinician did not complete the study due to lack of clinical experience.

### 2.4 Procedure

Participants were initially educated on the HMD, including how to properly don headset and utilize controllers. After initial set up of HMD, study lead accessed the OVE on HMD, to allow the clinician to view application. Clinicians viewed optokinetic background with both pass through and fully immersive setting (Figure 4). Using the controllers, clinicians navigated menu features to adjust visual parameters including speed of lines (scale of 1–10), number of dots (1 or 2), distance between dots, dot depth, and speed of dots (scale of 1–10). Experience with the OVE was self-guided, and while time was not formally collected, participants spent between 5 and 15 min engaging with the application. HMDs were manually cleaned using disinfectant wipes between uses.

### 2.5 Data analysis

Semi-structured interviews were conducted with neurorehabilitation clinicians between October 2024 and March 2025 (Supplementary Table 1). All interviews were led by a neuro-physical therapy expert (KD). Interviews were recorded and transcribed. Discussions lasted approximately 30 min and included open-ended questions as well as pre-defined probes. The interview guide focused on three main areas: 1. Usability of the HMD-based application, 2. Approaches to improve the utility and experience of the OVE, and 3. Methods and barriers to integrating into existing clinical workflows.

**FIGURE 3:**
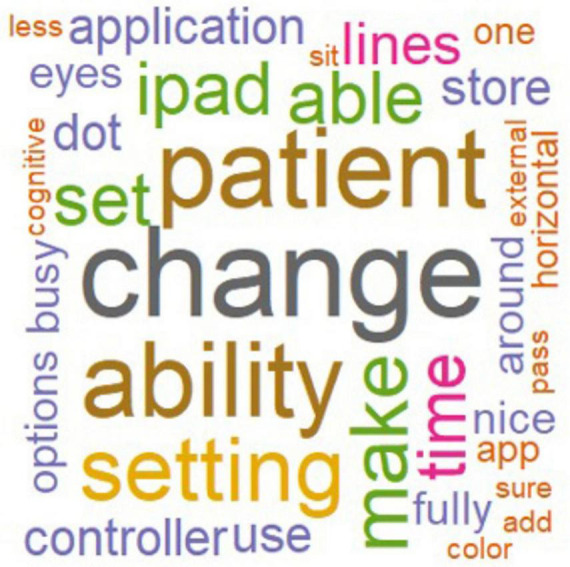
Word cloud representation of key terms identified in clinician interviews regarding the Optokinetic Virtual Environment (OVE) application. Frequently cited terms, such as *patient*, *change*, *ability*, and *setting*, appear larger, reflecting their higher frequency across responses. This visualization highlights clinicians’ emphasis on ability to adapt experiences, optimize clinicians’ ease of use, and incorporating patient-centered features.

A word cloud visualization (Figure 3) was generated using RStudio to display all words appearing more than once, with word size proportional to frequency and color indicating relative frequency across the data. Word-level preprocessing included conversion to lowercase, removal of punctuation (including dashes and quotation marks), common stop-words (e.g., “also,” “just,” “like,” “can”), and filtering out unique-instance terms.

Coding and theme identification were conducted using a reflexive thematic analysis approach as described by Braun and Clarke (39). Initial familiarization and open coding were independently performed by a physician-scientist with experience in neurorehabilitation and qualitative research (KS) and a neuro-physical therapist with vestibular expertise (KD) and a third team member (SS) reviewed coded transcripts to support consistency and completeness. Coding disagreements were addressed through iterative discussion until consensus was reached, with codes refined or merged as appropriate. This consensus-based approach emphasized shared interpretation rather than inter-rater reliability metrics, consistent with reflexive thematic analysis methodology. To support reflexivity, the analytic team met bi-monthly to discuss how professional backgrounds influence interpretation and generalizability. Notes were maintained during coding to document analytic decisions and evolving theme definitions. Final themes were defined, named, and reviewed collaboratively, and all themes were supported by representative participant quotations included in the Results and summary tables.

Thematic saturation was assessed iteratively during data collection and analysis. Saturation was considered achieved when successive interviews failed to generate novel codes or meaningfully alter existing themes. In this study, saturation was reached after eight interviews; two additional interviews were conducted and confirmed thematic stability, yielding no new codes or themes. To further illustrate coding outcomes, we have added representative excerpts and consolidated participant quotations within thematic tables, enabling readers to directly link raw data to analytic conclusions.

Generative AI (ChatGPT) was used to (1) assist with literature synthesis, (2) support organization and summarization of already-identified codes and themes, and (3) improve clarity and consistency of manuscript language. Importantly, AI tools were not used to generate primary codes, define themes independently, interpret raw interview transcripts, or make analytic decisions. All coding, theme development, and interpretive judgments were performed by the human research team. Outputs generated with AI assistance were reviewed, edited, and validated by the authors to ensure fidelity to the original data and to maintain analytic integrity.

## 3 Results

Table 1 displays participant demographics. Ten therapists used the OVE prototype and completed interviews. Thematic saturation was achieved at eight interviews. Two additional interviews were conducted without the emergence of additional themes.

**TABLE 1:** Participant demographics.

Characteristic	*N* = 10
Age in years	
Mean (SD)	34.5 (4.7)
Sex, n (%)	
Male	1 (10)
Female	9 (90)
Experience in years	
Mean (SD)	9 (4.4)
Range	3–15
Specialty, n (%)	
Occupational	2 (20)
Physical	8 (80)
Used VR previously, n (%)	4 (40)

### 3.1 Overall qualitative experience

**FIGURE 4:**
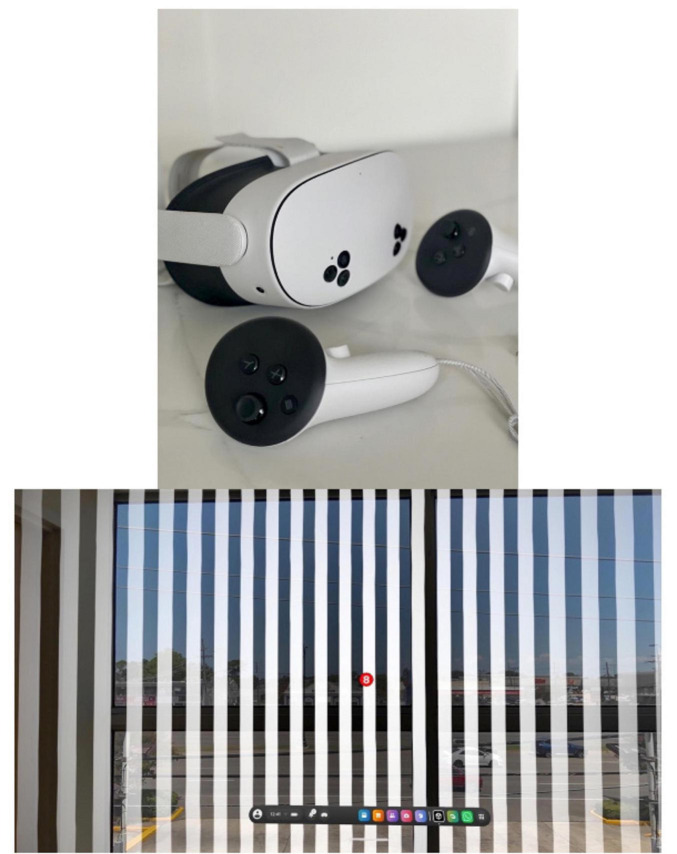
Top: meta quest 3 HMD bottom. Screen shot from HMD demonstrating optokinetic stimuli with pass-through enabled in a physical therapy gym.

Clinician participants expressed positive perceptions about using a medical extended reality application as an adjunct to vestibular therapy. Clinicians rated the likelihood of using the OVE in clinical practice as high, with Likert scores ranging from 6 to 10 (median = 9) with 10 representing highly likely. The primary potential limitation cited was variable patient tolerance. Of note, no clinician participants noted adverse effects, like vertigo or nausea, related to the use of the VR HMD (Figure 4, top).

### 3.2 Themes

**FIGURE 5:**
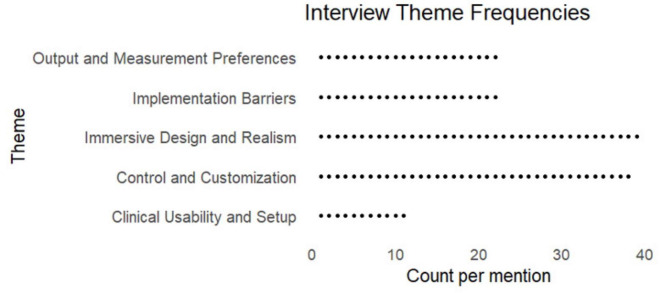
Dot plot of themes frequency. Dots represent individual coded references rather than unique participant counts. Participant counts may be found in the text.

The analysis revealed five overarching themes (Figure 5): (1) clinical usability and setup, (2) control and customization needs, (3) immersive design and realism, (4) output and measurement preferences, and (5) implementation barriers.

#### 3.2.1 Clinical usability and setup

Clinicians emphasized the importance of ease of setup, noting that while the application was “generally easy to set up,” some clinicians and patients may lack the technical proficiency to independently engage with an HMD-based experience. The need for education regarding the application and the HMD surfaced. Several providers (*n* = 5) suggested streamlining the initiation process by minimizing the steps required for setup to mitigate staff burden and reduce patient downtime. There were multiple mentions of improving clinic implementation (*n* = 7), with one participant suggesting incorporating formal training or orientation for interested clinicians.

*“Have [an] in-service and have people try it out, give them the time to play around with it.”* Participant 01, PT

#### 3.2.2 Control and customization needs

A prominent theme was the desire for therapist-directed control over settings, preferably via an external device like a tablet (*n* = 7). Clinicians highlighted the inconvenience of needing to remove the HMD mid-session to calibrate and adjust the settings of the OVE experience. The disruptive nature of stopping therapy for therapeutic modifications was noted to be a potential barrier to clinical operationalization. There were requests for preset standardized protocols like saccades (*n* = 3) as well as the ability to implement real-time modifications to protocols to optimize rehabilitation sessions (*n* = 4).

*“Put all settings onto one controller. Seems like it would make more sense for the therapist to be in control of the settings.”* Participant 10, OT

*“Therapist should be able to control and change settings mid-session.”* Participant 7, OT

#### 3.2.3 Immersive design and realism

Clinicians consistently valued the potential of immersive and realistic environments to mirror daily life scenarios (e.g., grocery stores, busy streets, escalators) (*n* = 8) as well as personalized experiences tailored to individual’s functional goals. Modern VR HMDs like the Meta Quest 3 have a “pass-through” capability where external-facing cameras transmit real-time video of the physical environment to the user within the headset. This feature enables users to maintain spatial orientation with the real-world environment while simultaneously engaging with virtual experiences without removing the headset (Figure 4, Bottom). Multiple clinician participants (*n* = 3) preferred the “pass-through” mode over full immersion for initial sessions, citing lower stimulation thresholds for sensitive patients. Others recommended more dynamic and visually complex scenes.

*“Pass through is less stimulation and fully immersive is more. I like the option to have both. I can start with the pass through and progress to fully immersive.”* Participant 02, PT

*“Eventually would like fully immersive scenarios like a busy grocery store, walking downtown, up/down escalator, a busy shopping mall to do visual scanning.”* Participant 09, PT

#### 3.2.4 Output and measurement preferences

Respondents (*n* = 5) noted interest in having clinically relevant metrics, including head movement speed, time tolerated, and dot velocity. Biometrics like pulse, respiratory rate, and heart rate variability were also mentioned as relevant metrics representative of clinical outcomes. Multiple participants (*n* = 3) mentioned the potential for integrating gamified elements (e.g., scoring, dot tracking) to encourage engagement, personalize treatment, and support progress monitoring.

*“Would be nice to have a summary of what we just did.”* Participant 10, OT

*“Would like time tolerated, speed of head movements, and speed of dot movement for smooth pursuits.”* Participant 04, PT

*“Would be nice to have more gamification, like getting a score.”* Participant 07, OT

#### 3.2.5 Implementation barriers

The most common concern mentioned was that patients would be unable to use and tolerate a VR HMD, especially among those with high motion sensitivity (*n* = 6). Additional impediments to clinical integration surfaced during interviews. Participants specifically mentioned concerns about clinic logistics such as cleaning protocols (*n* = 1), space for storing and charging equipment (*n* = 1), and coordinating device access (*n* = 1). Several respondents noted that the application was best suited to sitting activities and may be limited to open or shared spaces (*n* = 4).

*“Not all patients are tech savvy and able to use VR.”* Participant 01, PT

## 4 Discussion

The findings of this VR1-phase qualitative study provide clinician-derived design requirements for an optokinetic virtual environment intended to augment vestibular rehabilitation. Our findings align with the broader literature describing clinician interest in immersive VR approaches for vestibular rehabilitation and highlight design and workflow considerations that may influence adoption. This research also highlights persistent barriers to implementation that have limited routine clinical integration like usability and workflow fit, clinician control requirements, patient tolerance, and the need for clinically meaningful measurement outputs (32, 40). Importantly, as a VR1-phase qualitative study, these findings reflect clinician perceptions and design considerations rather than patient outcomes, validated usability metrics, or clinical efficacy.

### 4.1 Clinical usability and therapist control are prerequisites for implementation

There was general enthusiasm across participants to utilize VR in therapy consistent with literature on its use in rehabilitation (41). There, however, remains a general hesitancy of using VR due to concerns about whether this technology can effectively be operationalized in a busy clinic with minimal setup burden and minimal interruption to therapeutic flow. This emphasis is consistent with prior clinical VR literature describing how HMDs can introduce friction in actual clinical implementations through hardware management, required technical proficiency, and mid-session adjustments that disrupt therapy (42).

Accordingly, clinicians in our sample prioritized therapist-directed control (ideally externalized) to adjust stimulus parameters without removing the headset. This preference is also congruent with broader human factors considerations in rehabilitation technology: tools that preserve clinician agency and reduce session disruption are more likely to be adopted than those requiring frequent device interaction by the patient or clinician during care delivery (43).

### 4.2 HMD equipment can influence exercise performance through tolerance, comfort, and sensory load

Another key clinical concern raised by participants was patient usability and tolerance, particularly among individuals who already suffer from motion sensitivity. This concern is well supported by the cybersickness literature, where VR is associated with disorientation, nausea, and oculomotor symptoms in susceptible users, and where physiologic perturbations have been observed during cybersickness episodes (44).

Importantly, “exercise performance” in vestibular contexts is often constrained by symptom provocation thresholds rather than strength or endurance limitations alone. Thus, device factors like fit, weight, heat, sensory load, and the ability to grade exposure are particularly relevant in the use of VR in those with vestibular dysfunction. Seo et al. demonstrated that overall usability of VR HMDs will significantly impact whether a VR intervention can be delivered effectively. In parallel, the broader HMD balance literature suggests that head-mounted VR systems are increasingly capable for balance-related assessment and training (45), but that outcomes vary substantially by population, protocol, experiential complexity and device parameters which reinforces the need for staged validation that goes beyond early feasibility and focuses on usability (46–48).

### 4.3 Quantified summaries can bridge VR experiences to clinical reasoning and personalized treatment

Clinicians consistently requested session summaries and objective metrics like tolerance time, head movement speed, and stimulus velocity. This feedback reflects a need to translate a VR experience into the language of clinical evaluation and management. This aligns with a broader movement in rehabilitation toward instrumented feedback, where sensor-enabled systems support auditing of dose, adherence, and real-world activity patterns. For example, the SIRRACT trial demonstrated that wireless sensing could provide “ground truth” about patient activity patterns in inpatient stroke rehabilitation (49). Even though this additional feedback did not always translate into improved outcomes, it highlights that measurement infrastructure can still be clinically valuable for visibility and workflow integration. In vestibular rehabilitation, clinician-facing dashboards can serve as a practical bridge between immersive stimuli and standard clinical workflows by enabling rapid parameter adjustments, documentation support, and longitudinal progress tracking.

### 4.4 IMUs and real-time feedback can extend measurement and personalization for vestibular therapy

Participants’ interest in quantification and biofeedback is consistent with the wearable sensor literature describing the clinical utility of Inertial Measure Unit (IMUs). IMUs are devices that measure and report body specific forces, angular rates, and orientation. IMUs capture movement kinematics and support feedback-enabled rehabilitation paradigms (50). In vestibular-specific applications, IMU-guided systems have also been explored for real-time feedback on head rotation angles and speeds. IMUs have been able to determine peak head turning speeds in patients after vestibular schwannoma resection, with improvements in speed following vestibular rehabilitation (51). Additionally, IMUs have been used during positional maneuvers, supporting the concept that IMU-derived metrics can meaningfully augment vestibular care delivery when appropriately designed (52). Together, these data support the rationale for incorporating IMU-driven session summaries and therapist dashboards in future iterations of the OVE to better align with clinician expectations for actionable metrics that support patient-specific treatments.

### 4.5 Implementation barriers extend beyond software: infection control and clinic logistics

Beyond usability and tolerance, clinicians raised pragmatic concerns ranging from cleaning protocols, device storage/charging, and storage/space constraints. These issues are increasingly recognized as real adoption determinants for MXR. Recent literature has noted the absence and need for a standardized approach to disinfecting VR headsets (53). Addressing these basic logistical issues is likely necessary for successful real-world clinical deployment.

### 4.6 Limitations and implications for VR2/VR3 evaluation

This study is limited in generalizability due to a small sample size from a single institution that only included clinicians. Thematic saturation suggests, however, that a larger sample size would likely only yield incremental value. Inclusion of therapists from different sites or organizations would support external validation. Clinician participants were non-randomized, unblinded colleagues who may have influenced each other’s responses and introduced a diffusion of treatment effect as described by Urban (54).

### 4.7 Future directions

**FIGURE 6:**
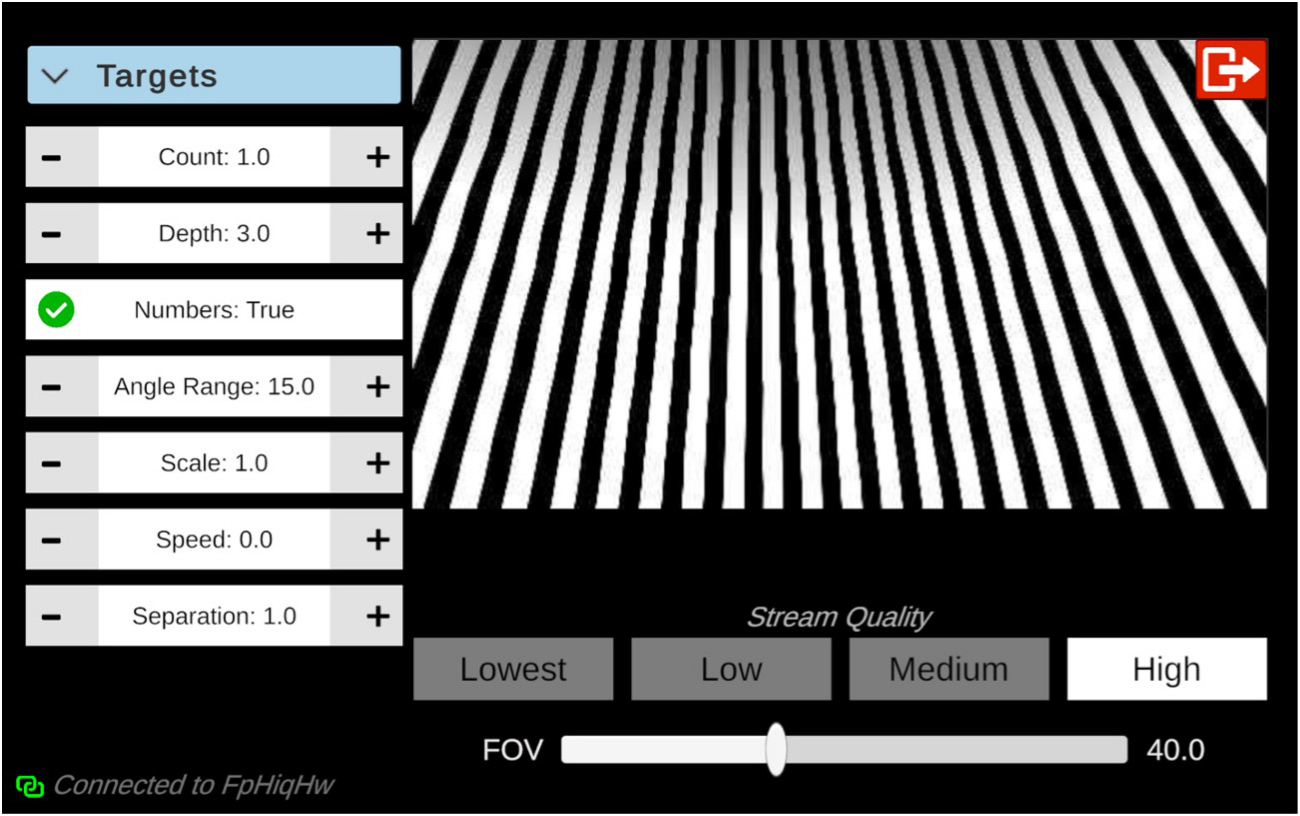
Proposed tablet-based operating system for the optokinetic virtual environment. Multiple parameters can be adjusted including number and velocity of fixation points, speed and spacing of optokinetic stimuli.

Participants’ feedback emphasized the need for streamlined controls, increased environmental variation including pass-through and realistic environments, enhanced quantification of sessions, and approaches to optimize clinical integration. Future iterations of the OVE will include a tablet-based interface focused on improving the clinician and patient experience (Figure 6). Future versions of the OVE will incorporate network code to support streaming of the HMD view to a tablet so that clinicians can observe the patient experience in real-time. The interface will also include graphic shaders allowing clinician-driven and dynamic adjustment of stimulus parameters such as speed, width, color, direction, and pattern type within a patient session. Clinicians will have the ability to save and restore stimulus settings to enable efficient clinical utilization and integration.

**FIGURE 7:**
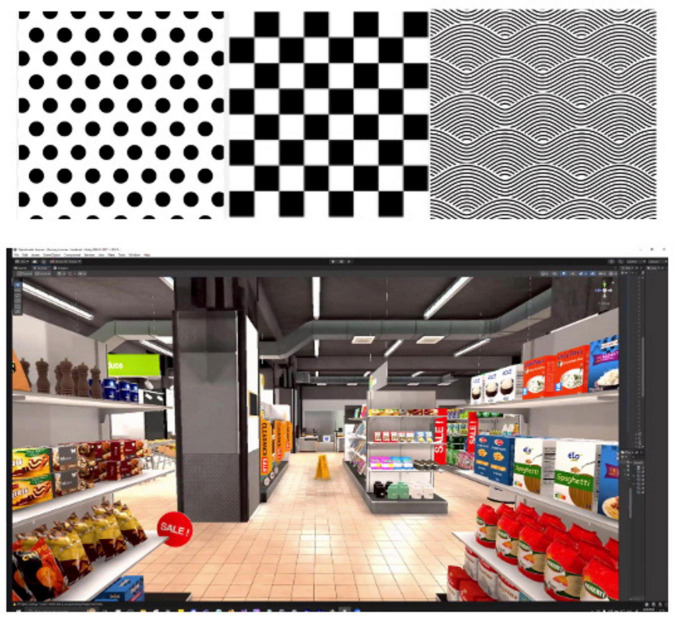
Examples of proposed additional environments. Top: different shaped optokinetic stimuli left to right: simple polka dot, checkerboard, and swirl background. Bottom: grocery store aisle.

To support real-world relevance and clinical generalizability, the visual environments will be expanded to include various abstract optokinetic stimuli that can be implemented with either complete immersion or pass-through, allowing for a blend of digital and physical realities. Future versions will also include immersive settings that mimic common dizziness triggers, such as grocery stores, cafes, and driving (Figure 7). Clinician-facing dashboards will be incorporated to provide session summaries and basic patient performance metrics such as time-used and repetitions performed. Future iterations may explore integration of eye-tracking and wearable sensors to support exploratory measurement of physiological and behavioral signals in subsequent usability and efficacy studies. Additionally, implementation barriers like clinical space needs, charging, and cleaning HMDs will be addressed.

Future studies will involve collecting feedback from a larger and more diverse cohort of clinicians as well as vestibular therapy patients. This will inform a finalized OVE application whose safety, tolerance, and efficacy will be evaluated in a randomized controlled clinical trial as consistent with VR2 and VR3 of the VR-CORE framework. Spending adequate time and effort in VR1 to address clinician and patient experience feedback is necessary prior to progressing to VR2 investigations. In addition, addressing implementation barriers will optimize future implementation studies of VR-supported vestibular rehabilitation across diverse care settings, including under-resourced clinics and remote rehabilitation scenarios.

## 5 Conclusion

The FDA has recognized MXR as a new avenue for clinical treatment, including interventions for neurorehabilitation. Neurorehabilitation MXR interventions should be developed using the framework developed by the VR Clinical Outcomes Research Experts (VR-CORE) committee (31). This qualitative study focused on the process and findings of VR1 for an MXR intervention to augment vestibular therapy. Results demonstrate that vestibular rehabilitation specialists expressed a willingness to consider an immersive OVE as a potential adjunct to traditional therapy, contingent on usability, workflow fit, and patient tolerance. This group endorsed a willingness to utilize VR-based optokinetic stimuli to enhance engagement, simulate real-world challenges, and individualize vestibular treatment. The analysis identified five actionable themes: clinical usability and setup, control and customization, immersive realism, output and feedback metrics, and implementation barriers. These themes offer clear guidance for future iterations of the OVE that will support translational, ethical, and scalable MXR neuro-rehabilitation solutions. These findings underscore the value of early end-user engagement in MXR tool development, particularly within neurorehabilitation.

## Data Availability

The raw data supporting the conclusions of this article will be made available by the authors, without undue reservation.

## References

[1] Steinmetz JD Seeher KM Schiess N Nichols E Cao B Servili C Global, regional, and national burden of disorders affecting the nervous system, 1990–2021: a systematic analysis for the Global Burden of Disease Study 2021. *Lancet Neurol.* (2024) 23:344–81. 10.1016/s1474-4422(24)00038-3 38493795 PMC10949203

[2] Harris E. Neurological conditions are leading cause of disability worldwide. *JAMA.* (2024) 331:1440. 10.1001/jama.2024.5160 38607619

[3] Lei J Gillespie K. Projected Global Burden of Brain Disorders Through 2050 (P7-15.001). *Neurology.* (2024) 102:3234. 10.1212/WNL.0000000000205009

[4] Langhorne P Bernhardt J Kwakkel G. Stroke rehabilitation. *Lancet.* (2011) 377:1693–702. 10.1016/S0140-6736(11)60325-5 21571152

[5] Khan F Turner-Stokes L Ng L Kilpatrick T. Multidisciplinary rehabilitation for adults with multiple sclerosis. *Cochrane Database Syst Rev.* (2007) 2007:CD006036. 10.1002/14651858.CD006036.pub2 17443610 PMC8992048

[6] Radder DLM Lígia Silva de Lima A Domingos J Keus SHJ van Nimwegen M Bloem BR Physiotherapy in Parkinson’s disease: a meta-analysis of present treatment modalities. *Neurorehabil Neural Repair.* (2020) 34:871–80. 10.1177/1545968320952799 32917125 PMC7564288

[7] Izard SG Juanes JA García Peñalvo FJ Estella JMG Ledesma MJS Ruisoto P. Virtual reality as an educational and training tool for medicine. *J Med Syst.* (2018) 42:50. 10.1007/s10916-018-0900-2 29392522

[8] Le Bras A Boustia F Janot K Le Pabic E Ouvrard M Fougerou-Leurent C Rehearsals using patient-specific 3D-printed aneurysm models for simulation of endovascular embolization of complex intracranial aneurysms: 3d SIM study. *J Neuroradiol.* (2023) 50:86–92. 10.1016/j.neurad.2021.11.008 34914933

[9] Ernst M Folkerts AK Gollan R Lieker E Caro-Valenzuela J Adams A Physical exercise for people with Parkinson’s disease: a systematic review and network meta-analysis. *Cochrane Database Syst Rev.* (2023) 1:CD013856. 10.1002/14651858.CD013856.pub2 36602886 PMC9815433

[10] Bahar-Fuchs A Clare L Woods B. Cognitive training and cognitive rehabilitation for mild to moderate Alzheimer’s disease and vascular dementia. *Cochrane Database Syst Rev.* (2013) 2013:CD003260. 10.1002/14651858.CD003260.pub2 23740535 PMC7144738

[11] Etoom M Khraiwesh Y Lena F Hawamdeh M Hawamdeh Z Centonze D Effectiveness of physiotherapy interventions on spasticity in people with multiple sclerosis: a systematic review and meta-analysis. *Am J Phys Med Rehabil.* (2018) 97:793–807. 10.1097/PHM.0000000000000970 29794531

[12] Sherrington C Fairhall NJ Wallbank GK Tiedemann A Michaleff ZA Howard K Exercise for preventing falls in older people living in the community. *Cochrane Database Syst Rev.* (2019) 1:CD012424. 10.1002/14651858.CD012424.pub2 30703272 PMC6360922

[13] Dee M Lennon O O’Sullivan C. A systematic review of physical rehabilitation interventions for stroke in low and lower-middle income countries. *Disabil Rehabil.* (2020) 42:473–501. 10.1080/09638288.2018.1501617 30508495

[14] Saumur TM Gregor S Xiong Y Unger J. Quantifying the amount of physical rehabilitation received by individuals living with neurological conditions in the community: a scoping review. *BMC Health Serv Res.* (2022) 22:349. 10.1186/s12913-022-07754-4 35296315 PMC8925183

[15] Saluja A Dhamija RK. Prioritizing neuro-rehabilitation services in low-and middle-income countries: needs, challenges and possible solutions. *Ann Indian Acad Neurol.* (2022) 25:579–82. 10.4103/aian.aian_499_22 36211136 PMC9540924

[16] Pryde SJ Williams O O’Hare MP Murdock C Pedlow K. Exploring access to community neurorehabilitation for people with progressive neurological conditions: a qualitative study. *Disabil Rehabil.* (2025) 47:142–55. 10.1080/09638288.2024.2338198 38632940

[17] Triki CC Leonardi M Mallouli SZ Cacciatore M Karlshoej KC Magnani FG Global survey on disruption and mitigation of neurological services during COVID-19: the perspective of global international neurological patients and scientific associations. *J Neurol.* (2022) 269:26–38. 10.1007/s00415-021-10641-3 34117527 PMC8195244

[18] Yin P Gao Y Chen R Liu W He C Hao J Temperature-related death burden of various neurodegenerative diseases under climate warming: a nationwide modelling study. *Nat Commun.* (2023) 14:8236. 10.1038/s41467-023-44066-5 38086884 PMC10716387

[19] Alt Murphy M Pradhan S Levin MF Hancock NJ. Uptake of technology for neurorehabilitation in clinical practice: a scoping review. *Phys Ther.* (2023) 104:zad140. 10.1093/ptj/pzad140 37856528 PMC10851848

[20] Strupp M Dieterich M Brandt T. The treatment and natural course of peripheral and central vertigo. *Dtsch Arztebl Int.* (2013) 110:505-15; quiz 515-6. 10.3238/arztebl.2013.0505. 24000301 PMC3752584

[21] Corrales CE Bhattacharyya N. Dizziness and death: an imbalance in mortality. *Laryngoscope.* (2016) 126:2134–6. 10.1002/lary.25902 26865242

[22] Strupp M Dlugaiczyk J Ertl-Wagner BB Rujescu D Westhofen M Dieterich M. Vestibular disorders. *Dtsch Arztebl Int.* (2020) 117:300–10. 10.3238/arztebl.2020.0300 32530417 PMC7297064

[23] Hall CD Herdman SJ Whitney SL Anson ER Carender WJ Hoppes CW Vestibular rehabilitation for peripheral vestibular hypofunction: an updated clinical practice guideline from the academy of neurologic physical therapy of the american physical therapy association. *J Neurol Phys Ther.* (2022) 46:118–77. 10.1097/NPT.0000000000000382 34864777 PMC8920012

[24] Pavlou M Bronstein AM Davies RA. Randomized trial of supervised versus unsupervised optokinetic exercise in persons with peripheral vestibular disorders. *Neurorehabil Neural Repair.* (2013) 27:208–18. 10.1177/1545968312461715 23077146

[25] Hoppes CW Anson ER Carender WJ Marchetti GF Hall CD Whitney SL Type, dose, and outcomes of physical therapy interventions for unilateral peripheral vestibular hypofunction: protocol for a systematic review. *Syst Rev.* (2023) 12:164. 10.1186/s13643-023-02328-9 37710291 PMC10503155

[26] Han BI Song HS Kim JS. Vestibular rehabilitation therapy: review of indications, mechanisms, and key exercises. *J Clin Neurol.* (2011) 7:184–96. 10.3988/jcn.2011.7.4.184 22259614 PMC3259492

[27] Whitney SL Sparto PJ Cook JR Redfern MS Furman JM. Symptoms elicited in persons with vestibular dysfunction while performing gaze movements in optic flow environments. *J Vestib Res.* (2013) 23:51–60. 10.3233/VES-130466 23549055 PMC4880484

[28] Spiegel B. *VRx: How Virtual Therapeutics Will Revolutionize Medicine.* New York, NY: Basic Books (2020).

[29] Milgram P Kishino F. A taxonomy of mixed reality visual displays. *IEICE Trans Inform Syst.* (1994) 77:1321–9. doi: 10.1.1.102.4646

[30] Spiegel BMR Rizzo A Persky S Liran O Wiederhold B Woods S What is medical extended reality? A taxonomy defining the current breadth and depth of an evolving field. *J Med Ext Real.* (2024) 1:4–12. 10.1089/jmxr.2023.0012 38505474 PMC10945763

[31] Birckhead B Khalil C Liu X Conovitz S Rizzo A Danovitch I Recommendations for methodology of virtual reality clinical trials in health care by an international working group: iterative study. *JMIR Ment Health.* (2019) 6:e11973. 10.2196/11973 30702436 PMC6374734

[32] Xie M Zhou K Patro N Chan T Levin M Gupta MK Virtual reality for vestibular rehabilitation: a systematic review. *Otol Neurotol.* (2021) 42:967–77. 10.1097/MAO.0000000000003155 33782257

[33] Meldrum D Herdman S Vance R Murray D Malone K Duffy D Effectiveness of conventional versus virtual reality-based balance exercises in vestibular rehabilitation for unilateral peripheral vestibular loss: results of a randomized controlled trial. *Arch Phys Med Rehabil.* (2015) 96:1319–28.e1. 10.1016/j.apmr.2015.02.032. 25842051

[34] Ku J Kim YJ Cho S Lim T Lee HS Kang YJ. Three-Dimensional augmented reality system for balance and mobility rehabilitation in the elderly: a randomized controlled trial. *Cyberpsychol Behav Soc Netw.* (2019) 22:132–41. 10.1089/cyber.2018.0261 30596530

[35] Hazzaa NM Manzour AF Yahia E Mohamed Galal E. Effectiveness of virtual reality-based programs as vestibular rehabilitative therapy in peripheral vestibular dysfunction: a meta-analysis. *Eur Arch Otorhinolaryngol.* (2023) 280:3075–86. 10.1007/s00405-023-07911-3 36947249 PMC10220119

[36] Webster A Poyade M Coulter E Forrest L Paul L. Views of specialist clinicians and people with multiple sclerosis on upper limb impairment and the potential role of virtual reality in the rehabilitation of the upper limb in multiple sclerosis: focus group study. *JMIR Serious Games.* (2024) 12:e51508. 10.2196/51508 38669680 PMC11087863

[37] Schmid L Glässel A Schuster-Amft C. Therapists’ perspective on virtual reality training in patients after stroke: a qualitative study reporting focus group results from three hospitals. *Stroke Res Treat.* (2016) 2016:6210508. 10.1155/2016/6210508 28058130 PMC5183768

[38] Vasileiou K Barnett J Thorpe S Young T. Characterising and justifying sample size sufficiency in interview-based studies: systematic analysis of qualitative health research over a 15-year period. *BMC Med Res Methodol.* (2018) 18:148. 10.1186/s12874-018-0594-7 30463515 PMC6249736

[39] Braun V Clarke V. Using thematic analysis in psychology. *Qual Res Psychol.* (2006) 3:77–101. 10.1191/1478088706qp063oa 32100154

[40] Heffernan A Abdelmalek M Nunez DA. Virtual and augmented reality in the vestibular rehabilitation of peripheral vestibular disorders: systematic review and meta-analysis. *Sci Rep.* (2021) 11:17843. 10.1038/s41598-021-97370-9 34497323 PMC8426502

[41] Bateni H Carruthers J Mohan R Pishva S. Use of virtual reality in physical therapy as an intervention and diagnostic tool. *Rehabil Res Pract.* (2024) 2024:1122286. 10.1155/2024/1122286 38304610 PMC10834096

[42] Garrett B Taverner T Gromala D Tao G Cordingley E Sun C. Virtual reality clinical research: promises and challenges. *JMIR Serious Games.* (2018) 6:e10839. 10.2196/10839 30333096 PMC6231864

[43] Mitchell J Shirota C Clanchy K. Factors that influence the adoption of rehabilitation technologies: a multi-disciplinary qualitative exploration. *J Neuroeng Rehabil.* (2023) 20:80. 10.1186/s12984-023-01194-9 37340496 PMC10280872

[44] Simón-Vicente L Rodríguez-Cano S Delgado-Benito V Ausín-Villaverde V Cubo Delgado E. Cybersickness. a systematic literature review of adverse effects related to virtual reality. *Neurologia.* (2024) 39:701–9. 10.1016/j.nrleng.2022.04.007 39396266

[45] Micarelli A Viziano A Augimeri I Micarelli D Alessandrini M. Three-dimensional head-mounted gaming task procedure maximizes effects of vestibular rehabilitation in unilateral vestibular hypofunction: a randomized controlled pilot trial. *Int J Rehabil Res.* (2017) 40:325–32. 10.1097/MRR.0000000000000244 28723718

[46] Soltani P Andrade R. The influence of virtual reality head-mounted displays on balance outcomes and training paradigms: a systematic review. *Front Sports Act Living.* (2020) 2:531535. 10.3389/fspor.2020.531535 33634259 PMC7902044

[47] Seo NJ Arun Kumar J Hur P Crocher V Motawar B Lakshminarayanan K. Usability evaluation of low-cost virtual reality hand and arm rehabilitation games. *J Rehabil Res Dev.* (2016) 53:321–34. 10.1682/JRRD.2015.03.0045 27271199 PMC6710625

[48] Ersin K Gürlek E Güler H Kalaycık Ertugay Ç Şerbetçioğlu MB. Appropriate image selection with virtual reality in vestibular rehabilitation: cross-sectional study. *JMIR Serious Games.* (2023) 11:e40806. 10.2196/40806 37052976 PMC10162482

[49] Dorsch AK Thomas S Xu X Kaiser W Dobkin BH. SIRRACT: an international randomized clinical trial of activity feedback during inpatient stroke rehabilitation enabled by wireless sensing. *Neurorehabil Neural Repair.* (2015) 29:407–15. 10.1177/1545968314550369 25261154 PMC4375021

[50] Porciuncula F Roto AV Kumar D Davis I Roy S Walsh CJ Wearable movement sensors for rehabilitation: a focused review of technological and clinical advances. *PM R.* (2018) 10:S220–32. 10.1016/j.pmrj.2018.06.013 30269807 PMC6700726

[51] Weston AR Dibble LE Fino P Lisonbee R Hoppes C Loyd BJ. Recovery of turning speed in patients after vestibular schwannoma resection. *J Vestib Res.* (2024) 34:145–57. 10.3233/VES-230097 38669501

[52] Pastor CAC Kwon C Joo JS Kim HC Shim DB Ku Y Feasibility of an inertial measurement unit sensor-based guiding system for benign paroxysmal positional vertigo treatment: a pilot study. *Sci Rep.* (2023) 13:3169. 10.1038/s41598-023-29685-8 36823440 PMC9950366

[53] Murray TS Shenoy ES Roberts SC Martinello R Marks A Hieftje K Applying principles to practice: cleaning and disinfection of extended reality equipment used in healthcare settings. *Infect Control Hosp Epidemiol.* (2025) 46:1–3. 10.1017/ice.2025.10249 40826908 PMC12483620

[54] Urban JB van Eeden-Moorefield BM. *Designing and Proposing Your Research Project.* Washington, DC: American Psychological Association (2018).

