# Supplementary material for: Design and preliminary evaluation of a VR-based optokinetic stimuli system for vestibular rehabilitation: insights from clinical end users
Source: Front Med (Lausanne). 2026 Mar 4;13:1766706. doi: 10.3389/fmed.2026.1766706 (PMC12995672; doi:10.3389/fmed.2026.1766706)
Supplement: Supplementary file 1 [file Table_1.docx]

**Optokinetic VR Expert Opinion**

1. On a scale of 0 to 10, would you use this application to treat patients with vestibular impairments?

0 1 2 3 4 5 6 7 8 9 10

Not at all Very Likely

1. In what ways would you improve this application to make it more user friendly in the clinic?
   1. Set up of application and HMD
   2. Ability to control the settings
   3. View of application on iPad
   4. Anything else?
2. Is there any information that you would like to have from the application that is not already provided?
   1. Display of output variables
   2. Measurements not already on menu

Good measure s

- 1. Other?

1. What are potential barriers to using the HMD and this application in the clinic?
